# Concomitant Treatment with Voriconazole and Flucloxacillin: A Combination to Avoid

**DOI:** 10.3390/antibiotics10091112

**Published:** 2021-09-15

**Authors:** Ruth Van Daele, Joost Wauters, Pieter De Cock, Franky Buyle, John Leys, Pieter Van Brantegem, Matthias Gijsen, Pieter Annaert, Yves Debaveye, Katrien Lagrou, Willy E. Peetermans, Roger J. Brüggemann, Isabel Spriet

**Affiliations:** 1 Department of Pharmaceutical and Pharmacological Sciences, KU Leuven, 3000 Leuven, Belgium; pieter.vanbrantegem@kuleuven.be (P.V.B.); matthias.gijsen@uzleuven.be (M.G.); pieter.annaert@kuleuven.be (P.A.); isabel.spriet@uzleuven.be (I.S.); 2 Pharmacy Department, University Hospitals Leuven, 3000 Leuven, Belgium; 3 Department of Microbiology, Immunology and Transplantation, KU Leuven, 3000 Leuven, Belgium; joost.wauters@uzleuven.be (J.W.); katrien.lagrou@uzleuven.be (K.L.); willy.peetermans@uzleuven.be (W.E.P.); 4 Medical Intensive Care Unit, University Hospitals Leuven, 3000 Leuven, Belgium; 5 Department of Basic and Applied Medical Sciences, Ghent University, 9000 Ghent, Belgium; pieter.decock@uzgent.be; 6 Department of Pharmacy, Ghent University Hospital, 9000 Ghent, Belgium; franky.buyle@uzgent.be; 7 Department of Pediatric Intensive Care, Ghent University Hospital, 9000 Ghent, Belgium; 8 Department of Pharmacy, Antwerp University Hospital, 2650 Edegem, Belgium; john.leys@uza.be; 9 Department of Cellular and Molecular Medicine, KU Leuven, 3000 Leuven, Belgium; yves.debaveye@uzleuven.be; 10 Intensive Care Unit, University Hospitals Leuven, 3000 Leuven, Belgium; 11 Clinical Department of Laboratory Medicine and National Reference Centre for Mycosis, Excellence Centre for Medical Mycology (ECMM), University Hospitals Leuven, 3000 Leuven, Belgium; 12 Department of Internal Medicine, University Hospitals Leuven, 3000 Leuven, Belgium; 13 Department of Pharmacy and Radboud Institute for Health Sciences, Radboudumc, 6525 GA Nijmegen, The Netherlands; roger.bruggemann@radboudumc.nl; 14 Radboudumc Center for Infectious Diseases, Radboudumc, 6525 GA Nijmegen, The Netherlands

**Keywords:** drug–drug interaction, voriconazole, flucloxacillin, CYP450 enzymes, underexposure

## Abstract

Background: Voriconazole is an antifungal drug used as one of the first-line treatments for invasive aspergillosis. This drug is extensively metabolized, predominantly via cytochrome P450 enzymes. An interaction between flucloxacillin and voriconazole, leading to subtherapeutic voriconazole concentrations, has previously been reported. We aimed to demonstrate that flucloxacillin independently influences voriconazole exposure. Methods: Patients from three Belgian hospitals, treated with a combination of voriconazole and flucloxacillin, were included in this retrospective study. Voriconazole concentrations were collected both in a timeframe with and without flucloxacillin co-treatment. Multivariate analyses were performed to study the independent effect of flucloxacillin treatment on voriconazole exposure and the possible influence of the flucloxacillin dose. Results: Thirty-three patients were included in this study and 145 trough concentrations (51 with, and 94 without concomitant flucloxacillin treatment) were analyzed. The median (IQR) voriconazole trough concentration sampled during flucloxacillin co-treatment was 0.5 (0–1.8) mg/L, whereas samples without flucloxacillin co-treatment had a median (IQR) voriconazole trough concentration of 3.5 (1.7–5.1) mg/L (*p* = 0.002), while receiving similar voriconazole doses. Subtherapeutic concentrations (<1 mg/L) were observed in 69% and 7% of the samples with flucloxacillin co-treatment versus samples without flucloxacillin co-treatment, respectively. Conclusion: This study shows that flucloxacillin co-treatment independently decreases voriconazole exposure. Caution is needed when these two drugs are administered simultaneously.

## 1. Introduction

Voriconazole is an antifungal drug with a longstanding history as a first-line treatment for invasive aspergillosis [1]. This drug is extensively metabolized, predominantly via the cytochrome P450 (CYP450) enzyme, CYP2C19, and to a lesser extent, via CYP2C9 and CYP3A4 [2]. In vitro studies suggest that the flavin-containing monooxygenases (FMOs), FMO3 and FMO1, also contribute to voriconazole metabolism [3]. Voriconazole is bound to plasma proteins for 58%, and is renally excreted as an unchanged drug for less than 2% [2,4].

As a clear exposure–response relationship has been established for voriconazole, it is important to attain an adequate exposure within the target range. A voriconazole trough concentration (C_min_) above 1–2 mg/L is associated with an improved efficacy [5,6], and so recommended in international guidelines with a higher target (>2 mg/L) for the treatment of fungi with an elevated minimum inhibitory concentration (MIC) value, or diseases with a poor prognosis [7,8]. The upper limit for voriconazole C_min_ was set to 5–6 mg/L [7,8], mostly based on the increased risk for neurotoxicity above this concentration [5]. Exposure to voriconazole is influenced by many factors, such as non-linear pharmacokinetics (PK) due to a saturable metabolism, erratic absorption after oral administration, and its involvement in many drug–drug interactions (DDIs), leading to a high intrapatient and interpatient variability, and the need for therapeutic drug monitoring (TDM) [2,9,10].

Kennedy et al. [11] first published a case report describing a possible interaction between voriconazole and flucloxacillin. They reported a sudden drop in voriconazole exposure after the initiation of flucloxacillin and subtherapeutic voriconazole concentrations were measured, despite dose augmentation, until flucloxacillin therapy was ceased [11]. Similar findings were reported in a retrospective study (*n* = 20) by Muilwijk et al. [12] in which subtherapeutic voriconazole concentrations (<1 mg/L) were observed in 11/20 patients receiving voriconazole and flucloxacillin simultaneously. The mechanism behind this interaction has not yet been elucidated. An activation of the pregnane X (PXR) receptor by flucloxacillin with an induction of the CYP450-enzymes expression has been suggested [11,13,14,15,16]; however, this mechanism is not supported in all published studies [12].

Based on previous descriptive reports, it seems that flucloxacillin may decrease voriconazole exposure; however, this has only been reported in one case report and one retrospective, multicenter study. The goal of this study is to confirm that the presence of flucloxacillin is independently associated with low voriconazole exposure.

## 2. Results

### 2.1. Patients

Thirty-three patients were included in this study with a median (interquartile range (IQR)) age of 59 (50–70) years, body weight of 59 (55–73) kg, and a length of hospital stay of 33 (14–52) days. Most patients (88%) were treated with voriconazole for (possible or probable) invasive aspergillosis. None of the patients received ECMO or CYP450-inducers during the study period.

### 2.2. Voriconazole Concentrations

During voriconazole therapy, a total of 235 samples were collected. Two sample sets were constructed to exclude incorrect trough concentrations. Sample set A consisted of the actual voriconazole concentrations, analyzed as a continuous variable, only including trough concentrations that were collected 12 h ± 1 h after the previously administered dose (*n* = 128). In sample set B, voriconazole concentrations were categorized as a binary variable, i.e., in subtherapeutic and (supra)therapeutic concentrations (to assess target attainment). The latter was carried out by including the voriconazole concentrations from sample set A, along with the subtherapeutic concentrations that were collected too early, or therapeutic concentrations that were collected too late (*n* = 145), since interpretation of these concentrations would not have changed in the case of a correct sampling time.

In 12 patients (36%, sample set A), voriconazole concentrations were collected both in a timeframe before and during flucloxacillin treatment (Table S4). The median (IQR) drop in voriconazole concentration (between the last concentration before flucloxacillin initiation and the first concentration under flucloxacillin) in these patients was −1.15 ((−2.05)–0.03) mg/L. During flucloxacillin treatment, the voriconazole dose was increased in 15 out of 30 evaluable patients.

As shown in Table 1, the median (IQR) voriconazole trough concentration was 0.5 (0–1.8) mg/L for the samples collected during flucloxacillin co-treatment, and 3.5 (1.7–5.1) mg/L for those without flucloxacillin co-treatment (*p* = 0.002), while receiving a similar voriconazole dose (*p* = 0.54). Subtherapeutic concentrations below 1 mg/L were observed in 69% and 7% of the samples with flucloxacillin co-treatment versus samples without flucloxacillin co-treatment, respectively. When the lower limit of 2 mg/L was used, subtherapeutic concentrations were observed in 78% (with flucloxacillin) and 27% (without flucloxacillin) of the samples.

In the subset of therapeutic voriconazole C_min_ (>1 mg/L) under flucloxacillin co-treatment (*n* = 16, Table 2), the median (IQR) C_min_ was 2.3 (1.8–4.8) mg/L.

Further details on both voriconazole and flucloxacillin treatment are presented in Table 1. More information on the administered voriconazole and flucloxacillin therapy, and the combination with voriconazole, can be found in Supplementary Materials Tables S1–S3.

**Figure 1:**
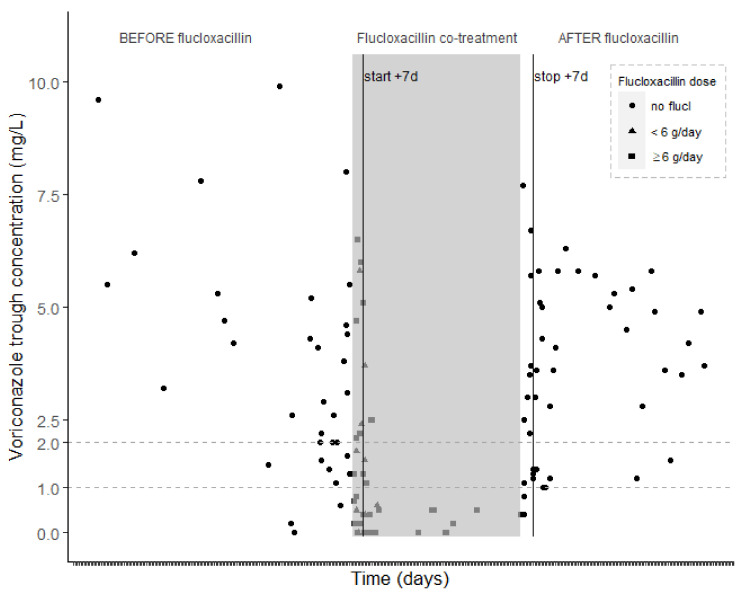
Voriconazole concentration as a function of time before, during, and after association of flucloxacillin. The grey area represents the time period in which flucloxacillin was administered in combination with voriconazole. The white areas are the periods of time of voriconazole administration before and after flucloxacillin therapy, respectively. Each break on the x-axis represents one day and is depicted relative to the start and stop of flucloxacillin administration. Black dots: voriconazole concentrations without flucloxacillin; grey triangle/square: voriconazole concentrations while treated with flucloxacillin, depending on the administered dose. The black vertical solid lines illustrate the 7th day after the initiation and the stop of flucloxacillin.

The voriconazole concentration in a timeframe before, during, and after flucloxacillin treatment is depicted in Figure 1 (C_min_), Figure S1 (C_min_ corrected for administered dose), and Figure S2 (C_min_ in the subgroup of patients with voriconazole C_min_ in both a period with and without the flucloxacillin combination), respectively.

### 2.3. Multivariate Analysis

In the multivariate analysis, the influence of different covariates (flucloxacillin co-treatment, voriconazole dose, day of voriconazole treatment, mode of voriconazole administration, co-treatment with proton pump inhibitors (PPIs), and the use of continuous renal replacement therapy (CRRT)) on the voriconazole concentrations was explored. In the final model, which was built on samples for which all included covariates were known or estimated (*n* = 102), flucloxacillin co-treatment was included as a significant covariate leading to lower voriconazole concentrations (beta: −1.92), along with the oral route of administration (beta: −1.14). A higher voriconazole dose (beta: 0.14) and the association of PPIs (beta: 1.40) were significantly associated with higher voriconazole concentrations. In the subset of samples collected under flucloxacillin treatment (*n* = 44), the flucloxacillin dose was also included in the final model after backward selection, with a higher flucloxacillin dose leading to lower voriconazole concentrations (beta: −0.18). The voriconazole concentration as a function of the administered flucloxacillin dose is shown in Table S3, and the different flucloxacillin doses are depicted in Figure 1 and Figure S1.

## 3. Discussion

In this multicenter retrospective study, flucloxacillin co-treatment was found to be an independent predictor of decreased voriconazole exposure.

A lower voriconazole exposure and a higher proportion of subtherapeutic trough concentrations were observed in the voriconazole samples with the combination of flucloxacillin compared to samples without the combination, despite a similar voriconazole daily dose. These results were supported by the multivariate analysis, in which flucloxacillin co-treatment was retained as a significant covariate associated with decreased voriconazole trough concentrations. Moreover, in Figure 1, a clear drop in voriconazole concentrations is shown during flucloxacillin administration. These results are in line with the previous case report of Kennedy et al. [11] and the retrospective study of Muilwijk et al. [12]. In the latter study, subtherapeutic concentrations (i.e., <1 mg/L) during flucloxacillin therapy were observed in 11/20 (55%) of the patients [12]. In our study, an even higher proportion was observed; 24/31 (77%) of the patients had at least one C_min_ under flucloxacillin below 1 mg/L and 26/31 (84%) below 2 mg/L.

In vitro studies reported that the flucloxacillin-mediated CYP450 enzyme induction is concentration-dependent [13,14]. In our study, the flucloxacillin dose was also significantly associated with the voriconazole C_min_. However, in the study of Du et al. [16], the interaction was already observed at a dose of 500 mg twice daily and Muilwijk et al. [12] reported an effect of flucloxacillin independent of the administered dose. It is plausible that the interaction will only take place with the high, and frequently used, dosing regimens of 6 or 12 g/day, or that the magnitude of the interaction is more pronounced with these higher doses, but further studies should explore if this interaction also takes place with lower flucloxacillin doses.

Our study has shown that flucloxacillin independently reduces voriconazole exposure. It is uncertain if augmented voriconazole doses lead to an adequate exposure during flucloxacillin therapy. The data in this study were collected during routine therapeutic drug monitoring, and reflect real-life clinical practice, in which subtherapeutic concentrations were frequently observed despite increased voriconazole doses in many patients (~50%).

The underlying mechanistic explanation for this interaction is unknown. Activation of the PXR receptor with an induction of the CYP450 enzymes expression has been suggested [11]. This hypothesis is supported by in vitro studies observing an upregulation of CYP450-enzymes via PXR activation by flucloxacillin [13,14]. Moreover, a similar induction interaction with flucloxacillin has previously been reported with repaglinide and tacrolimus, both metabolized via CYP450-enzymes [15,16]. The typical time delay associated with PXR-mediated induction, with a maximum induction effect occurring only after 1–2 weeks of flucloxacillin and a similar delay after flucloxacillin cessation [17], was also confirmed in the study with tacrolimus [15]. Based on the results in our study (Figure 1), a delay in the onset and cessation of the flucloxacillin effect was indeed observed. In contrast with the previous studies, doubts about the PXR mechanism of action were raised by Muilwijk et al. [12], who observed an instantaneous (median of 2 days) onset and cessation of the interaction upon the association and stop of flucloxacillin. Moreover, in this study, it was reported that other drugs metabolized by CYP3A4 (such as cyclosporine and tacrolimus) were unaffected. As suggested by Veenhof et al., the latter might be explained by the fact that voriconazole is a CYP3A4 inhibitor which opposes the inducing effect of flucloxacillin, or by frequent dose adjustments based on therapeutic drug monitoring, e.g., tacrolimus and cyclosporine [15]. Induction via PXR is a probable mechanism behind this studied interaction, but this should be confirmed for voriconazole in in vitro experiments. Other mechanistic explanations, that may be considered in the case that the PXR hypothesis could not be confirmed, and that may explain low voriconazole concentrations without a PXR-associated delay-time as observed by Muilwijk et al., are a shift toward the FMO3 metabolism pathway [3] or post-transcriptional effects on CYP450 enzymes, such as impact on the stability of CYP450 enzymes (as already observed by the stabilization of CYP2E1 by ethanol) [18], regulation at the level of mRNA stability [18], or direct CYP450 activation.

Although the majority of voriconazole C_min_ were subtherapeutic under flucloxacillin therapy, a minority were still therapeutic and in a few patients, no subtherapeutic C_min_ were measured under flucloxacillin therapy (Table 2). This may be attributed to the fact that the maximum induction potential was not yet reached at the moment of sampling, since the median flucloxacillin treatment duration for the moment of sampling for these concentrations was only 6 (4–8) days. However, this may also indicate that there is a mechanistic underlying reason that makes some patients less sensitive to flucloxacillin induction, e.g., the genotype of the patient. Du et al. already suggested that the inductive activity of flucloxacillin in the interaction with repaglinide is genotype-dependent since an impaired PXR activity, associated with PXR single-nucleotide polymorphisms (SNPs) −298G/G and 11193C/C, leading to a lower expression of CYP3A4/2C8, may make PXR less sensitive to being induced [16].

An important limitation of this study is the retrospective design. To ensure a correct interpretation of the results, samples that were not collected as correct trough concentration were excluded from this study. Due to the retrospective design of our study, some covariates (such as liver function parameters) were missing in a large part of the dataset and could not be used as covariates in the multivariate analysis. Second, no daily TDM was performed in clinical practice, so the exact time frame for the onset or stop of this interaction is difficult to define. However, pooling all collected samples gives a good distribution of concentrations over time. Third, drug–drug interactions were considered, but only at the moment of voriconazole therapy, and not before voriconazole initiation, which may be relevant for induction reactions. Moreover, as a CYP2C19 inhibitor, only the frequently used PPIs were included. Finally, flucloxacillin concentrations were not routinely collected in clinical practice; therefore, only the influence of the flucloxacillin dose, instead of flucloxacillin exposure, was studied.

In clinical practice, a switch to another antibiotic (e.g., cefazolin, clindamycin, or vancomycin) or antifungal (e.g., liposomal amphotericin B, or echinocandins) drug is recommended to avoid concomitant therapy with voriconazole and flucloxacillin. Assuming PXR induction as the underlying mechanistic explanation, it is important to take into account that other azoles may also be impacted by flucloxacillin. Isavuconazole is also metabolized via CYP3A4 and may theoretically be impacted by flucloxacillin. In contrast to voriconazole and isavuconazole, posaconazole is only metabolized for ~15%, predominantly via UDP-glucuronosyltransferase (UGT) 1A4 and not via CYP450 enzymes. Posaconazole is also a substrate for P-glycoprotein (P-gp) [19]. Since PXR is also involved in the expression of UGT1A4 and P-gp [20,21], flucloxacillin might also influence posaconazole concentrations. No reports have been published concerning the impact of flucloxacillin on isavuconazole and posaconazole concentrations, but TDM should be performed when these azoles are combined with flucloxacillin. If a switch in antibiotic or antifungal drugs is not possible, voriconazole concentrations should be strictly monitored during flucloxacillin therapy and a second antifungal drug should be initiated until therapeutic drug concentrations are reached.

Although multiple reports about the induction potential of flucloxacillin have been published, this interaction is not yet incorporated in most international drug–drug interaction checkers, nor in the summary of product characteristics of voriconazole, which may have led to insufficient awareness of this interaction and, consequently, underexposure to voriconazole in patients who are co-treated with flucloxacillin. It is possible that this interaction will also take place in combination with other CYP450-substrates, as has already been suggested for repaglinide and tacrolimus.

## 4. Materials and Methods

### 4.1. Study Design, Population and Setting

This retrospective study was performed in three Belgian hospitals: the University Hospitals Leuven (UZL); the Ghent University Hospital; and the Antwerp University Hospital, between January 2010 and January 2021. Every adult patient treated with the combination of voriconazole and flucloxacillin was eligible for this study if at least one voriconazole concentration was collected during this drug combination. There was no restriction as a function of indication or prescribed dose.

### 4.2. Data Collection

The following information was collected: patient demographics (gender, age, body weight, length of hospital stay); liver function parameters (bilirubin, gamma glutamyltransferase, alkaline phosphatase, alanine transaminase and aspartate transaminase); treatment with extracorporeal circuits (CRRT, intermittent hemodialysis (IHD) or extracorporeal membrane oxygenation (ECMO)); voriconazole and flucloxacillin dosing details (timing, administration and dosing information, voriconazole trough concentrations); and the concomitant administration of interacting drugs (rifampicin, rifabutin, phenytoin, phenobarbital, carbamazepine, St. John’s wort, antiretrovirals (cytochrome P 450 (CYP450)-inducers) and PPIs (CYP2C19 inhibition)).

### 4.3. Voriconazole Measured Concentrations

The collected voriconazole concentrations were only evaluable as actual trough concentrations if they were collected 12 h ± 1 h after the previously administered dose (sample set A). However, subtherapeutic concentrations (<1 mg/L) that were collected too early (<11 h after previous dose) or therapeutic concentrations (>1 mg/L) that were collected too late (>13 h after previous dose but before the next dose), were included for calculating the amount of subtherapeutic concentrations (sample set B), since classification of these concentrations would not have changed in the case of a correct sampling time. Since most guidelines recommend a target trough concentration of 1–2 mg/L, the proportion of concentrations below 2 mg/L was also reported.

The voriconazole concentrations were categorized into 2 groups, i.e., a group with and without flucloxacillin association. The first group (with flucloxacillin) consisted of voriconazole concentrations that were collected after at least one flucloxacillin dose was administered until 12 h after the cessation of flucloxacillin. The control group (without flucloxacillin) contained voriconazole trough concentrations collected in the same patients before flucloxacillin initiation or 12 h after flucloxacillin cessation.

### 4.4. Statistical Analysis

To compare the voriconazole trough concentrations and voriconazole doses in the sample groups with the flucloxacillin combination versus without the flucloxacillin combination, univariate generalized estimating equation (GEE) analyses were used, with the association of flucloxacillin as a binomial outcome variable. Moreover, a multivariate GEE analysis was performed to assess if the combination with flucloxacillin independently influenced voriconazole trough concentration (sample set A, continuous outcome variable). The following covariates were included in the multivariate model: flucloxacillin co-treatment (within 12 h before voriconazole sampling); the dose of voriconazole (mg/kg in the previous 24 h interval); the day of voriconazole treatment; the mode of voriconazole administration (intravenous or oral); the co-treatment with PPIs; and the use of CRRT. A backward selection was performed until a final model with only significant (*p*-value < 0.05) parameters was attained, or until flucloxacillin co-treatment was excluded from the model as a non-significant parameter.

To assess the influence of the flucloxacillin dose, a similar multivariate analysis was performed, but the predictor ‘flucloxacillin co-treatment’ was replaced by the ‘flucloxacillin dose’. The latter analysis was only performed in the subgroup of the samples that were collected under flucloxacillin treatment, which reduced the sample size for this analysis (*n* = 44). Consequently, to lower the risk of overfitting, the number of included predictors was decreased by the exclusion of CRRT as a covariate.

For statistical analysis, missing continuous data were completed with the median value for the same patient if available, or with the median of the total population otherwise. The multivariate analyses were performed with the voriconazole trough concentrations for which a value for all included covariates was available (collected or estimated using the median value). Graphs were created and statistical analyses were carried out with R statistics (R version 3.6.3; The R Foundation for Statistical Computing, Vienna, Austria).

## 5. Conclusions

In conclusion, our retrospective multicenter study showed that co-treatment with flucloxacillin independently decreases voriconazole concentrations, leading to subtherapeutic voriconazole exposure. Caution is needed when these two drugs are administered simultaneously. Flucloxacillin administration should be considered as contra-indicated in patients treated with voriconazole for invasive fungal infections. When the combination cannot be avoided, we recommend adding a second antifungal agent as long as therapeutic voriconazole concentrations are not warranted.

## Figures and Tables

**Table 1:** Voriconazole trough concentrations.

	Total	During Flucloxacillin Co-Treatment	Without Flucloxacillin Co-Treatment	*p*-Value
Continuous voriconazole trough concentrations of SAMPLE SET A ^a^				
Number of C_min_	128	45	83	NA
Trough concentration (mg/L), median (IQR)	2.2 (0.8–4.5)	0.5 (0–1.8)	3.5 (1.7–5.1)	0.002
Previous daily dose (mg/kg), median (IQR)	7.4 (5.3–10.2)	7.9 (6.8–11.0)	7.3 (5.3–9.6)	0.54
Mode of administration, IV, *n* (%)	40 (31)	20 (44)	20 (24)	0.19
Day of flucloxacillin therapy, median (IQR)	NA	8 (4–13)	NA	NA
Categorical voriconazole trough concentrations of SAMPLE SET B ^b^				
Number of C_min_	145	51	94	NA
Subtherapeutic C_min_ (<1 mg/L), *n* (%)	42 (29)	35 (69)	7 (7)	<0.0001
Subtherapeutic C_min_ (<2 mg/L), *n* (%)	65 (45)	40 (78)	25 (27)	0.0001
Previous daily dose (mg/kg), median (IQR)	7.3 (5.3–9.6)	7.9 (6.8–10.7)	7.3 (5.3–8.5)	0.79
Mode of administration, IV, *n* (%)	49 (34)	22 (43)	27 (29)	0.01
Number of patients with at least one subtherapeutic (<1 mg/L) C_min_, ratio *	24/33	24/31	5/22	NA
Number of patients with at least one subtherapeutic (<2 mg/L) C_min_, ratio *	27/33	26/31	15/22	NA
Day of flucloxacillin therapy, median (IQR)	NA	8 (5–12)	NA	NA

C_min_: trough concentration; *n*: number of samples; IQR: interquartile range; IV: intravenous. ^a^ Sample set A was built using actual voriconazole trough concentrations collected 12 h ± 1 h after the previous administered dose (continuous variable); ^b^ sample set B was built using concentrations included in sample set A along with subtherapeutic concentrations (<1 mg/L) which were collected too early (<11 h after previous dose) and (supra)therapeutic concentrations (>1 mg/L) which were collected too late (>13 h after previous dose), since interpretation of these concentrations would not have changed in the case of a correct sampling time (binary variable). * In 31 patients, a C_min_ was collected under simultaneous voriconazole and flucloxacillin therapy (cfr. Table S4).

**Table 2:** Therapeutic voriconazole trough concentrations under flucloxacillin therapy.

Therapeutic C_min_ under Flucloxacillin Therapy	C_min_ > 1 mg/L	C_min_ > 2 mg/L
Number of therapeutic C_min_ under flucloxacillin, *n* (%)	16 (31)	11 (22)
Trough concentration (mg/L), median (IQR)	2.3 (1.8-4.8)	3.7 (2.3–5.5)
Previous daily dose (mg/kg), median (IQR)	8.0 (7.2–11.4)	7.5 (7.0–9.7)
Day of flucloxacillin therapy, median (IQR)	6 (4–8)	6 (4–7)
Dose was previously increased under flucloxacillin association, (yes), *n* (%)	2 (13)	0 (0)
Number of patients with only therapeutic C_min_ under flucloxacillin, ratio *	7/31	5/31

C_min_: trough concentration; *n*: number. * In 31 patients, a C_min_ could be collected under simultaneous voriconazole and flucloxacillin therapy (cfr. Table S4).

## Data Availability

Individual participant data that underlie the results reported in this article are available from the corresponding author upon reasonable request, providing the request meets local ethical and research governance criteria after publication. Patient-level data will be anonymized and study documents will be redacted to protect the privacy of participants.

## Supplementary Materials

The following are available online at https://www.mdpi.com/article/10.3390/antibiotics10091112/s1, Figure S1: Voriconazole concentrations, corrected for the administered dose, as a function of time and association of flucloxacillin, Figure S2: Voriconazole concentrations in the subgroup of patients with voriconazole Cmin in both a period with and without flucloxacillin combination inf function of time, Table S1: Voriconazole administration, Table S2: Flucloxacillin administration, Table S3: Influence of flucloxacillin dose, Table S4: Voriconazole and flucloxacillin co-administration.

## References

[1] Ullmann A.J., Aguado J.M., Arikan-Akdagli S., Denning D.W., Groll A.H., Lagrou K., Lass-Florl C., Lewis R.E., Munoz P.E., Verweij F. (2018). Diagnosis and management of Aspergillus diseases: Executive summary of the 2017 ESCMID-ECMM-ERS guideline. Clin. Microbiol. Infect..

[2] Bellmann R., Smuszkiewicz P. (2017). Pharmacokinetics of antifungal drugs: Practical implications for optimized treatment of patients. Infection.

[3] Yanni S.B., Annaert P.P., Augustijns P., Bridges A., Gao Y., Benjamin D.K., Thakker D.R. (2008). Role of flavin-containing monooxygenase in oxidative metabolism of voriconazole by human liver microsomes. Drug Metab. Dispos..

[4] Pfizer (2021). Summary of Product Characteristics (SmPC) Vfend.

[5] Pascual A., Calandra T., Bolay S., Buclin T., Bille J., Marchetti O. (2008). Voriconazole therapeutic drug monitoring in patients with invasive mycoses improves efficacy and safety outcomes. Clin. Infect. Diss..

[6] Miyakis S., van Hal S.J., Ray J., Marriott D. (2010). Voriconazole concentrations and outcome of invasive fungal infections. Clin. Microbiol. Infect..

[7] Lewis R., Brüggemann R., Padoin C., Maertens J., Marchetti O., Groll A., Johnson E., Arendrup M. (2015). Triazole Antifungal Therapeutic Drug Monitoring; ECIL 6 Meeting. https://www.ebmt.org/sites/default/files/migration_legacy_files/document/ECIL%206-Triazole-TDM-07-12-2015-Lewis-R-et-al.pdf.

[8] Ashbee H.R., Barnes R.A., Johnson E.M., Richardson M.D., Gorton R., Hope W.W. (2014). Therapeutic drug monitoring (TDM) of antifungal agents: Guidelines from the British Society for Medical Mycology. J. Antimicrob. Chemother..

[9] Patterson T.F., Thompson G.R., Denning D.W., Fishman J.A., Hadley S., Herbrecht R., Kontoyiannis D.P., Marr K.A., Morrison V.A., Nguyen M.H. (2016). Practice Guidelines for the Diagnosis and Management of Aspergillosis: 2016 Update by the Infectious Diseases Society of America. Clini. Infect. Dis..

[10] Schulz J., Kluwe F., Mikus G., Michelet R., Kloft C. (2019). Novel insights into the complex pharmacokinetics of voriconazole: A review of its metabolism. Drug Metab. Rev..

[11] Kennedy B., Larcombe R., Chaptini C., Gordon D.L. (2015). Interaction between voriconazole and flucloxacillin during treatment of disseminated Scedosporium apiospermum infection. J. Antimicrob. Chemother..

[12] Muilwijk E.W., Dekkers B.G.J., Henriet S.S.V., Verweij P.E., Witjes B., Lashof A., Groeneveld G.H., van der Hoeven J., Alffenaar J.W.C., Russel F.G.M. (2017). Flucloxacillin Results in Suboptimal Plasma Voriconazole Concentrations. Antimicrob. Agents Chemother..

[13] Huwyler J., Wright M.B., Gutmann H., Drewe J. (2006). Induction of cytochrome P450 3A4 and P-glycoprotein by the isoxazolyl-penicillin antibiotic flucloxacillin. Curr. Drug Metab..

[14] Stage T.B., Graff M., Wong S., Rasmussen L.L., Nielsen F., Pottegård A., Brøsen K., Kroetz D.L., Khojasteh S.C., Damkier P. (2018). Dicloxacillin induces CYP2C19, CYP2C9 and CYP3A4 in vivo and in vitro. Br. J. Clin. Pharmacol..

[15] Veenhof H., Schouw H.M., Besouw M.T.P., Touw D.J., Gracchi V. (2020). Flucloxacillin decreases tacrolimus blood trough levels: A single-center retrospective cohort study. Eur. J. Clin. Pharmacol..

[16] Du Q.-Q., Wang Z.-J., He L., Jiang X.-H., Wang L. (2013). PXR polymorphisms and their impact on pharmacokinetics/pharmacodynamics of repaglinide in healthy Chinese volunteers. Eur. J. Clin. Pharmacol..

[17] Spriet I., Meersseman W., de Hoon J., von Winckelmann S., Wilmer A., Willems L. (2009). Mini-series: II. clinical aspects. clinically relevant CYP450-mediated drug interactions in the ICU. Intensiv. Care Med..

[18] Hakkola J., Hukkanen J., Turpeinen M., Pelkonen O. (2020). Inhibition and induction of CYP enzymes in humans: An update. Arch. Toxicol..

[19] Van Daele R., Spriet I., Maertens J. (2020). Posaconazole in prophylaxis and treatment of invasive fungal infections: A pharmacokinetic, pharmacodynamic and clinical evaluation. Expert Opin. Drug Metab. Toxicol..

[20] Liu W., Ramírez J., Gamazon E.R., Mirkov S., Chen P., Wu K., Sun C., Cox N.J., Cook E., Das S. (2014). Genetic factors affecting gene transcription and catalytic activity of UDP-glucuronosyltransferases in human liver. Hum. Mol. Genet..

[21] Synold T.W., Dussault I., Forman B.M. (2001). The orphan nuclear receptor SXR coordinately regulates drug metabolism and efflux. Nat. Med..

