# Supplementary material for: Concomitant Treatment with Voriconazole and Flucloxacillin: A Combination to Avoid
Source: Antibiotics (Basel). 2021 Sep 15;10(9):1112. doi: 10.3390/antibiotics10091112 (PMC8469006; doi:10.3390/antibiotics10091112)
Supplement: Supplementary file 1 [file antibiotics-10-01112-s001.zip › antibiotics-1377598-supplementary.pdf]

Concomitant treatment with voriconazole and flucloxacillin: a combination to avoid

Supplementary Materials

Table S1. Voriconazole administration (on moment of sampling)

SAMPLE SET A[a] (n=128)	
Voriconazole dose (mg/kg/day), *median [IQR]*	7.4 [5.3-10.2]
Voriconazole treatment day, *median [IQR]*	33 [11-88]
Mode of voriconazole administration, IV, *n (%)*	40 (31)
Total duration of voriconazole therapy, *median [IQR]* ($n_{patient}$=27)	43 [17-151]
SAMPLE SET B[b] (n=145)	
Voriconazole dose (mg/kg/day), *median [IQR]*	7.3 [5.3-9.6]
Voriconazole treatment day, *median [IQR]*	33 [11-90]
Mode of voriconazole administration, IV, *n (%)*	49 (34)
Total duration of voriconazole therapy, *median [IQR]* ($n_{patient}$=29)	43 [14-150]

n: number of samples; IQR: interquartile range; intravenous

[a] Sample set A was built using actual voriconazole trough concentrations collected 12h ±1h after the previous administered dose (continuous variable)

[b] Sample set B was built using concentrations included in sample set A along with subtherapeutic concentrations (< 1 mg/L) which were collected too early (<11h after previous dose) and (supra)therapeutic concentrations (> 1 mg/L) which were collected too late (>13h after previous dose), since interpretation of these concentrations would not have changed in case of correct sampling time (binary variable)

Table S2. Flucloxacillin administration (on moment of sampling)

SAMPLE SET A[a] (n=45)	
Flucloxacillin dose (g/day), *n(%)*	
0.5	1 (2)
2	3 (7)
3	2 (4)
4	4 (9)
6	11 (24)
12	24 (53)
Flucloxacillin dose (g/day), *median [IQR]*	12 [6-12]
Flucloxacillin treatment day, *median [IQR]*	8 [4-13]
Mode of flucloxacillin administration, IV, *n (%)* (n=42)	38 (91)
SAMPLE SET B[b] (n = 51)	
Flucloxacillin dose (g/day), *n(%)*	
0.5	1 (2)
2	3 (6)
3	2 (4)
4	5 (10)
6	11 (22)
12	29 (57)
Flucloxacillin dose (g/day), *median [IQR]*	12 [6-12]
Flucloxacillin treatment day on the moment of sampling, *median [IQR]*	8 [5-12]
Mode of flucloxacillin administration, IV, *n (%)* (n=47)	43 (92)

Cmin: trough concentration; n: number of samples; IQR: interquartile range; intravenous

[a] Sample set A was built using actual voriconazole trough concentrations collected 12h ±1h after the previous administered dose (continuous variable)

[b] Sample set B was built using concentrations included in sample set A along with subtherapeutic concentrations (< 1 mg/L) which were collected too early (<11h after previous dose) and (supra)therapeutic concentrations (> 1 mg/L) which were collected too late (>13h after previous dose), since interpretation of these concentrations would not have changed in case of correct sampling time (binary variable)

Table S3. Influence of flucloxacillin dose

Flucloxacillin dose (g/day)	n	Median [IQR] voriconazole concentration (mg/L)
0.5	1	2.2 [2.2-2.2]
2	3	0.4 [0.2-1.4]
3	2	1.2 [0.9-1.5]
4	4	2.7 [1.3-4.2]
6	11	0.4 [0.0-2.3]
12	24	0.4 [0.0-0.8]

n: number of voriconazole concentrations; IQR: interquartile range

Table S4. Voriconazole and flucloxacillin co-administration

Number of patients with voriconazole samples in the following periods of time, n(%)	
SAMPLE SET A[a]	
Only during flucloxacillin therapy	10 (30)
Before and during flucloxacillin therapy	6 (18)
During and after flucloxacillin therapy	6 (18)
Before, during and after flucloxacillin therapy	6 (18)
Only without flucloxacillin therapy	3 (9)
No concentrations in sample set A	2 (6)
SAMPLE SET B[b]	
Only during flucloxacillin therapy	10 (30)
Before and during flucloxacillin therapy	5 (15)
During and after flucloxacillin therapy	7 (21)
Before, during and after flucloxacillin therapy	9 (27)
Only without flucloxacillin therapy	2 (6)
**Number of patients with voriconazole and flucloxacillin therapy in the following sequence, n(%)**	
First voriconazole, flucloxacillin is added afterwards	17 (52)
First flucloxacillin, voriconazole is added afterwards	8 (24)
Simultaneous initiation of voriconazole and flucloxacillin	8 (24)

n: number of samples

[a] Sample set A was built using actual voriconazole trough concentrations collected 12h ±1h after the previous administered dose (continuous variable)

[b] Sample set B was built using concentrations included in sample set A along with subtherapeutic concentrations (< 1 mg/L) which were collected too early (<11h after previous dose) and (supra)therapeutic concentrations (> 1 mg/L) which were collected too late (>13h after previous dose), since interpretation of these concentrations would not have changed in case of correct sampling time (binary variable)

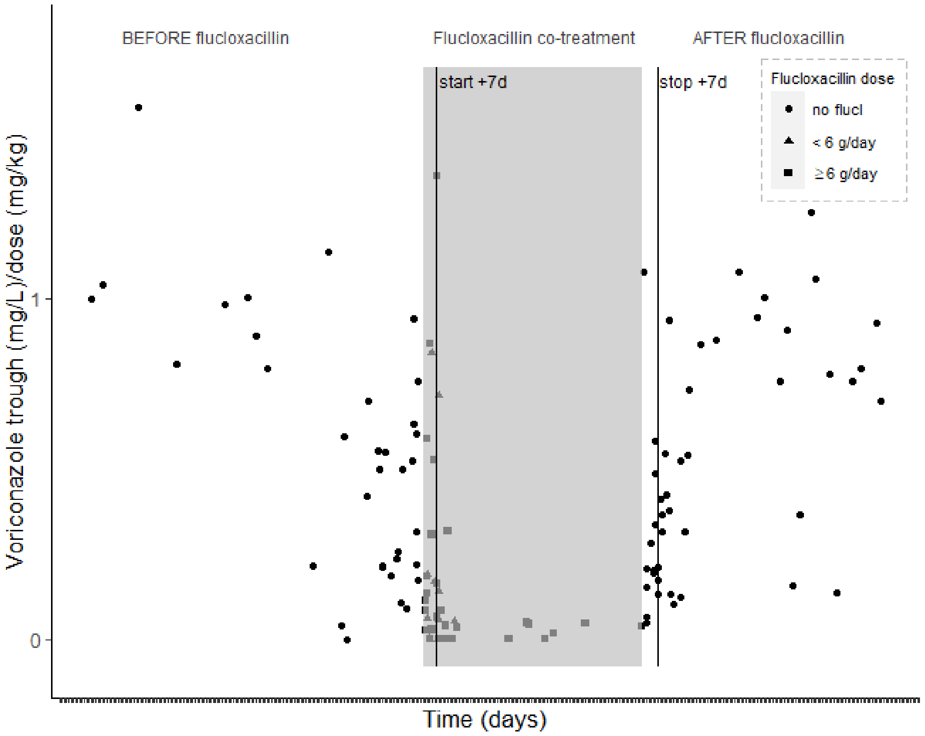

Figure S1. Voriconazole concentrations, corrected for the administered dose, in function of time, before during and after assocation of flucloxacillin. The grey area represents the time period in which flucloxacillin was administered in combination with voriconazole. The white area's are the periods of time of voriconazole administration before and after flucloxacillin therapy, respectively. Each break on the x-axis represents one day and is depicted relative to the start and stop of flucloxacillin administration. Black dots: voriconazole concentrations without flucloxacillin; grey triangle/square: voriconazole concentrations while treated with flucloxacillin, depending on the administered dose.
The black vertical solid lines illustrate the 7th day after initiation and stop of flucloxacillin.

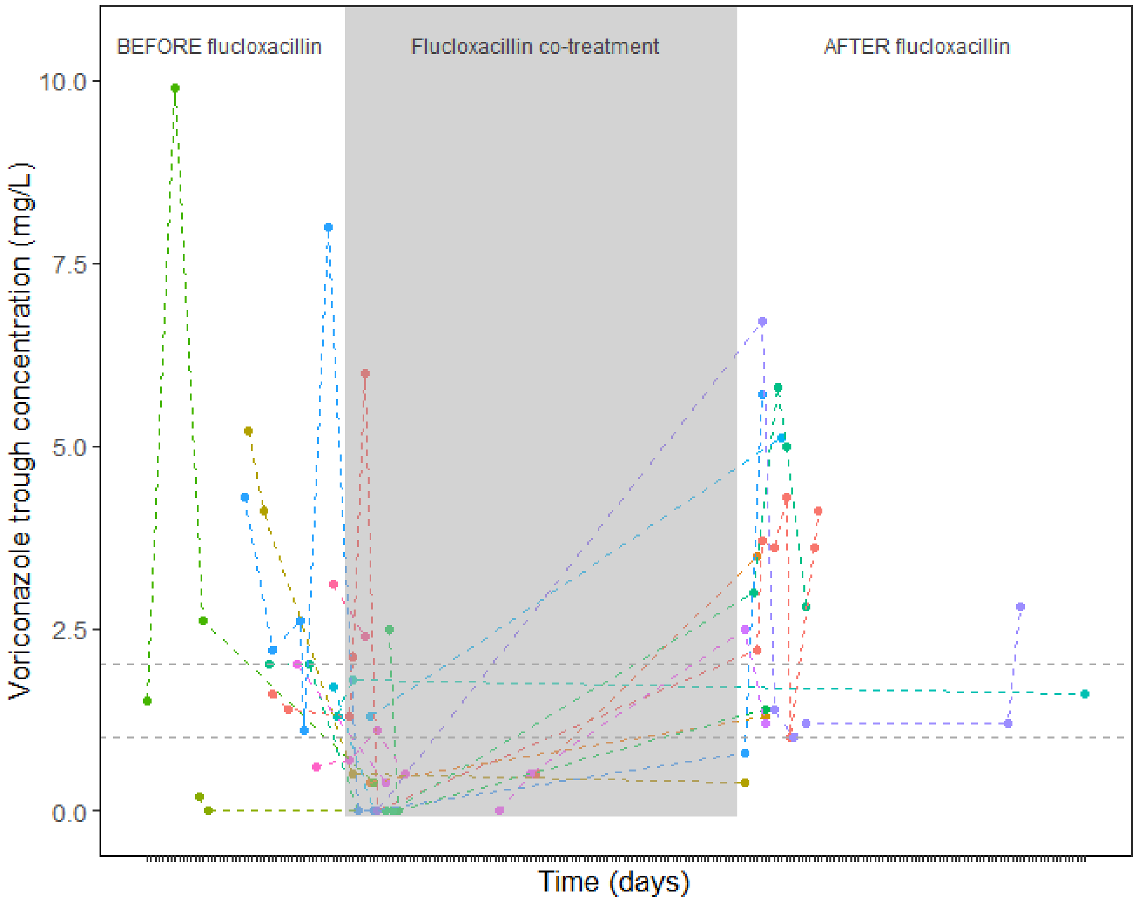

Figure S2. Voriconazole concentrations in function of time, before, during and after association of flucloxacillin for the patients with voriconazole C_{min} in both a period with and without flucloxacillin combination.

Different colours represent different patients and the dots the voriconazole concentrations.

The grey area represents the time period in which flucloxacillin was administered in combination with voriconazole. The white area's are the periods of time of voriconazole administration before and after flucloxacillin therapy, respectively. Each break on the x-axis represents one day and is depicted relative to the start and stop of flucloxacillin administration.
